# Synthesis of Pt nanoparticles and their burrowing into Si due to synergistic effects of ion beam energy losses

**DOI:** 10.3762/bjnano.5.197

**Published:** 2014-10-24

**Authors:** Pravin Kumar, Udai Bhan Singh, Kedar Mal, Sunil Ojha, Indra Sulania, Dinakar Kanjilal, Dinesh Singh, Vidya Nand Singh

**Affiliations:** 1 Inter University Accelerator Centre (IUAC), New Delhi 110067, India; 2 National Physical Laboratory (NPL), New Delhi 110012, India

**Keywords:** atomic force microscopy, burrowing of nanoparticles, medium-energy ion irradiation, nuclear and electronic energy loss, Rutherford backscattering spectroscopy, scanning electron microscopy, thin films, transmission electron microscopy

## Abstract

We report the synthesis of Pt nanoparticles and their burrowing into silicon upon irradiation of a Pt–Si thin film with medium-energy neon ions at constant fluence (1.0 × 10^17^ ions/cm^2^). Several values of medium-energy neon ions were chosen in order to vary the ratio of the electronic energy loss to the nuclear energy loss (*S*_e_/*S*_n_) from 1 to 10. The irradiated films were characterized using Rutherford backscattering spectroscopy (RBS), atomic force microscopy (AFM), scanning electron microscopy (SEM), X-ray diffraction (XRD) and high resolution transmission electron microscopy (HRTEM). A TEM image of a cross section of the film irradiated with *S*_e_/*S*_n_ = 1 shows ≈5 nm Pt NPs were buried up to ≈240 nm into the silicon. No silicide phase was detected in the XRD pattern of the film irradiated at the highest value of *S*_e_/*S*_n_. The synergistic effect of the energy losses of the ion beam (molten zones are produced by *S*_e_, and sputtering and local defects are produced by *S*_n_) leading to the synthesis and burrowing of Pt NPs is evidenced. The Pt NP synthesis mechanism and their burrowing into the silicon is discussed in detail.

## Introduction

The emergence of nanotechnology has opened up new research channels in almost every field of science [1–8]. The synthesis of nano-dimensional structures of various elements with narrow size distribution is a big challenge for scientists [9–11]. Due to certain advantages, namely, the control of growth parameters and spatial distribution, ion beam synthesis of buried nanoparticles (NPs) has received considerable attention in recent years [12–15]. The ions of desired elements, especially those of noble metals, are implanted into a matrix with certain fluence and post-annealing of the sample leads to the formation of NPs within the matrix. The energy, which governs the stopping of ions in matter, is chosen to obtain a desired particle distribution profile (longitudinal) in the matrix. The multiple energy implantations of the ions are used to increase this distribution profile further [16]. The transverse distribution is controlled by scanning the ion beam over the sample (desired matrix). The ion fluence and the annealing temperature are chosen to control the growth process leading to the final size distribution of the particles [17]. Homogenous nucleation requires a threshold concentration of implanted materials. Further, annealing may affect the spatial distribution of particles significantly due to thermally activated diffusion of implants.

Due to the fact that metallic ion beams produced from the electron cyclotron resonance ion source (ECRIS) (used in the current experiment) suffer from poor intensities and instabilities [18–19], a recently investigated novel method of synthesizing buried metallic NPs has been employed [20]. The ion irradiation of thin metallic films deposited on a suitable substrate (with lower surface energy) leads to the synthesis of metal NPs embedded into the substrate. The energy losses of the ions (not the ion itself) are mainly responsible for the resulting nano-structuring. The ion-induced point defects lead to the burrowing/diffusion of the surface NPs. The depth of the defect cascade can be more than the range. Only a few reports of such studies are available [21–26] and the exact mechanism of the formation of NPs is not very clear. The burrowing of self-organized cobalt clusters in a gold substrate upon thermal activation was reported by Padovani et al. [27]. When the surface energy of the metallic film is larger than that of the substrate, then surface nano-structuring is due to ion-induced sputtering of the film followed by the dewetting of metallic islands [20,28]. However, other effects such as ion-induced viscous flow, recoil implantation and thermodynamically driven capillary forces can also contribute to the formation of the buried NPs. When the ion beams with high electronic energy loss (dominates at high energies) pass through the material, a local melting (thermal spike) [29] occurs along the ion trajectory due to the energy deposition into the electronic subsystem (within 10^−16^ s). The local thermalization of the electronic sub-system takes place within 10^−14^ s. The deposited energy is transferred to the atomic subsystem by electron–phonon coupling. The melting of materials along the ion trajectory generates a surface tension gradient due to an imbalance of the surface and the interface energies, which further gives rise to mass transport through capillary action. The migration of metallic atoms and subsequent agglomeration can result in the formation of the nanoparticles. The ion trajectory formation in insulators and semiconductors after passage of high energy ions is mainly explained by the Coulomb explosion model [30]. However, ion beams with high nuclear energy loss (which dominates at low energies) in the materials undergo elastic scattering with the atoms of materials (for instance Pt and Si as in the present case), and finally, a collision cascade is achieved. Bolse [31] reported that a cylindrical local spike can be formed along a sub-cascade by the overlap of spherical thermal spikes.

To decouple the ion–matter interactions in the two types of energy loss processes and to better understand the synthesis mechanism of the NPs and their burrowing, neon ions of several energies were chosen. The interest in choosing Pt as the thin film was due to potential applications of Pt NPs [32–34]. Apart from their excellent catalytic performance, Pt NPs are used in fabricating super capacitors [35]. The Pt NPs in core–shell structures (Pt forms the shell) are used in surface enhanced Raman scattering (SERS) studies [36] as well. Moreover, Pt is relatively inert in atmosphere and ex situ characterization of irradiated samples can also be carried out. In this paper, we present the synthesis of Pt NPs and their burrowing in Si and discuss the possible mechanism.

## Experimental

Using thermal evaporation (deposition rate, 0.1 nm/s) under high vacuum conditions, 5 nm Pt thin films were deposited on a crystalline silicon substrate. The pressure inside the chamber before and during deposition was 2 × 10^−7^ and 3 × 10^−6^ mbar, respectively. The samples (Pt–Si, 10 × 10 mm) were irradiated using an ECRIS-based, upgraded version of the old low energy ion beam facility (LEIBF) [37] at IUAC, New Delhi. Ion irradiation was carried out in a vacuum chamber (≈10^−7^ mbar pressure) at normal incidence and at room temperature. All the samples were processed at an ion fluence of 1 × 10^17^ ions/cm^2^ with a constant beam current of 1 µA. The ion beam was scanned over the 15 × 15 mm area to achieve uniform irradiation conditions. The chosen beam energies for irradiation were 50, 140, 350 and 600 keV. For 350 keV and 600 keV ion irradiations, Ne^+2^ and Ne^+3^ ions were extracted from the ECR plasma and *E*/*q* values (the total potential difference including extraction and platform voltages) were set to 175 kV and 200 kV, respectively. The extraction of highly charged ions was employed to meet the energy requirements as accelerator operation with platform voltage beyond 250 kV was quite unsafe. Singly ionized neon ions were extracted for the irradiation of the films at other two energies and *E*/*q* values (50 kV for 50 keV, and 140 kV for 140 keV) were set accordingly. The irradiated samples were characterized using Rutherford backscattering spectroscopy (RBS), atomic force microscopy (AFM), scanning electron microscopy (SEM) and X-ray diffraction (XRD) techniques. High resolution cross sectional transmission electron microscopy (HRXTEM) of the sample irradiated with *S*_e_/*S*_n_ = 1 (where maximum burrowing was seen) was also performed in order to gain quantitative information, for example, particle size, depth of burrowing, etc. The morphological changes on the surfaces were studied using a multimode Nanoscope IIIa atomic force microscopy (AFM) in tapping mode. The AFM scans were made at a slower rate using a single crystal silicon tip. The apex of the tip has a radius of curvature of ≈10 nm and a locking frequency ≈350 KHz. For RBS measurements, 2 MeV He^+^ ions were bombarded onto the samples using the Pelletron Accelerator RBS-AMS System (PARAS) facility at IUAC, New Delhi. The backscattering yield was measured using a surface barrier detector mounted at 10º in the irradiation chamber with respect to the beam direction. The vacuum inside the chamber during irradiation was ≈10^−4^ Torr. He^+^ irradiation was carried out at 7° to avoid ion channeling in the samples. Before taking the spectra, an energy calibration was performed using the Au and Si edges (reference sample: Au deposited on the glass). For HRXTEM analysis, the sample was cut in 4 × 5 mm pieces using an ultrasonic disc cutter. These pieces were glued together (face-to-face and face-to-back) to form a cross. A 2.3 mm-diameter piece was drilled out (along the cross section) using an ultrasonic cutter. This piece was fixed (using epoxy) in a 3 mm-diameter brass tube. Thin slices were cut from this tube for mechanical thinning up to 100 µm. Then, the center of the slice was dimpled to achieve 20–30 µm thickness. The dimpled slice was ion milled to achieve final perforation and TEM analysis was performed. An X-ray diffractometer installed at IUAC was equipped with a conventional Cu K_α_ source, Göbble mirror, LiF monochromator, scintillator detector (NaI(Tl)) and was used to record the XRD pattern of pristine and irradiated films. For SEM measurements, a field emission scanning electron microscope (FE-SEM, MIRA II LMH from TESCAN) installed at IUAC with a resolution of 1.5 nm at 30 kV was used. This model has a secondary electron (SE) and a backscattered electron (BSE) detector for imaging.

## Results and Discussion

**Figure 1:**
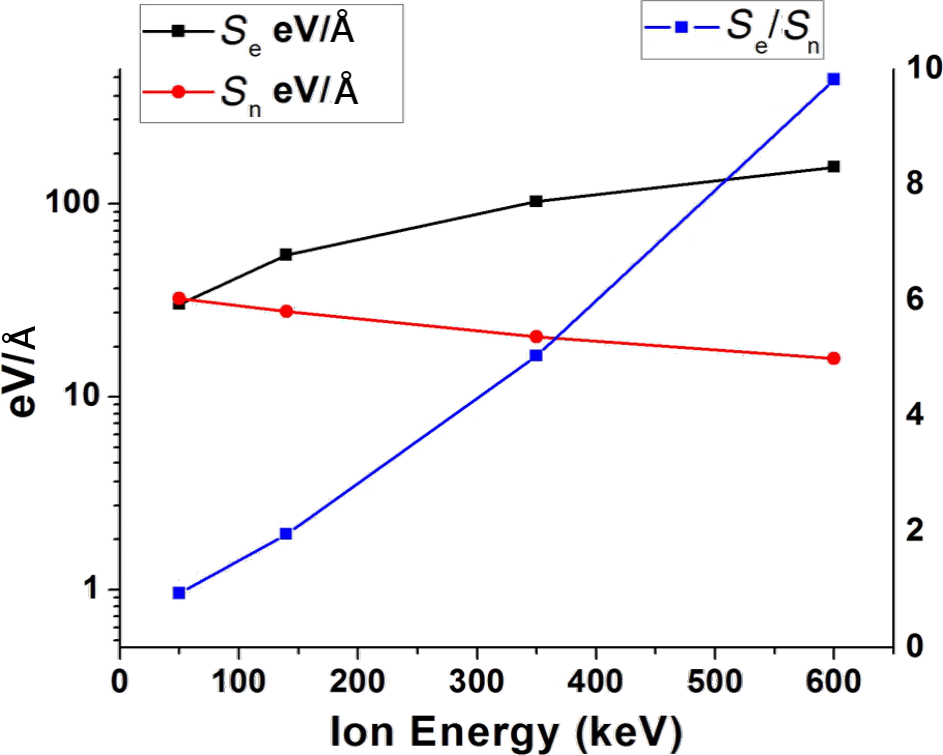
Electronic and nuclear stopping vs ion energy (SRIM calculation for neon ions incident on Pt).

Using stopping and range of ions in matter (SRIM) calculations [38], the energy losses (both electronic and nuclear) by neon ions in the Pt film as a function of ion energy is shown in Figure 1.

Unlike swift, heavy ions (with an energy of approximately hundreds of MeV) that undergo very high (on the order of keV/Å) electronic energy loss (*S*_e_) in the material, *S*_e_ by neon ions of chosen energies in Pt is quite low (≈155 eV/Å for 600 keV). For 50 keV neon ions, electronic (*S*_e_) and nuclear stopping (*S*_n_) are quite close to each other (≈30 eV/Å). With increasing energy, *S*_e_ increases and *S*_n_ decreases (*y* axis on the left hand side). The energy loss ratio, *S*_e_/*S*_n_, is also plotted as a function of ion energy for convenience (*y* axis on the right hand side).

**Figure 2:**
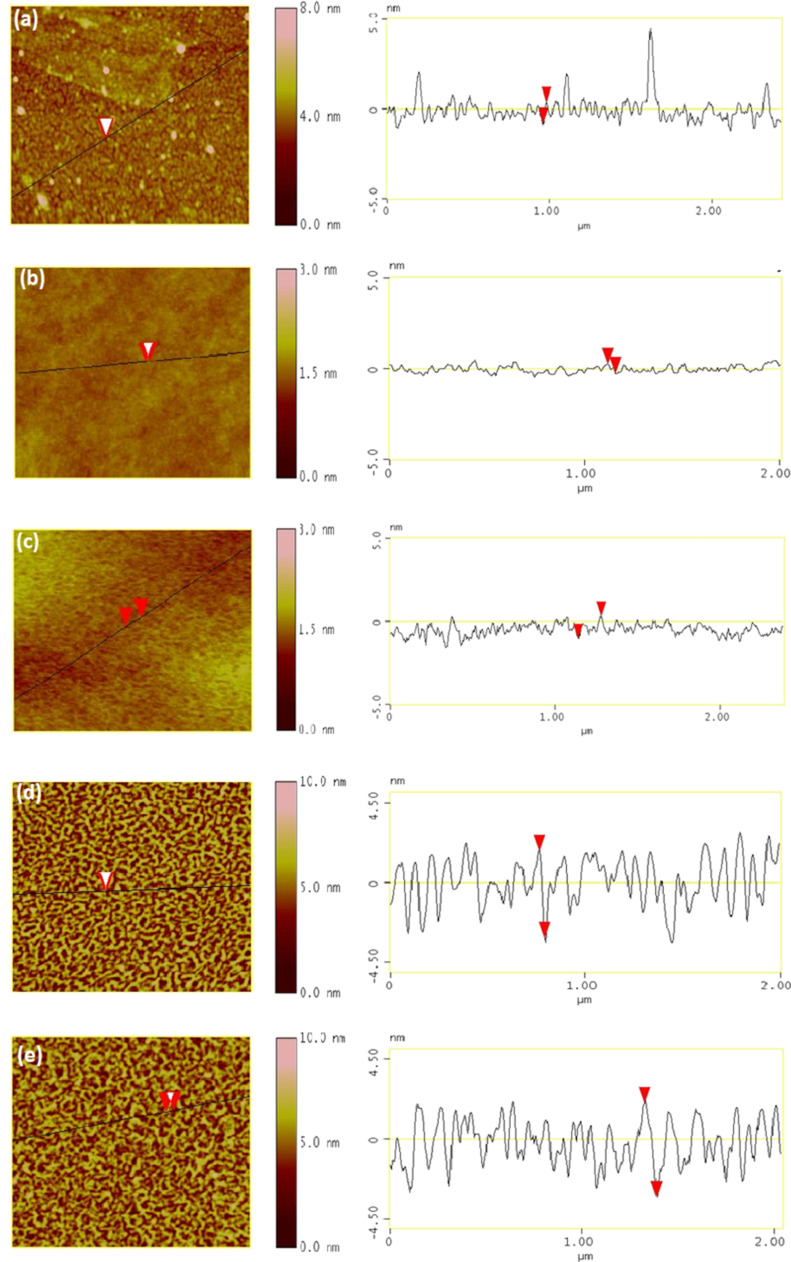
AFM images: a) pristine film, b–e) films irradiated with 50 keV, 140 keV, 350 keV and 600 keV, respectively.

The 2D surface morphology along with the sectional analysis (shown in the right hand side) of the pristine and ion-irradiated films deduced by AFM is shown in Figure 2a–e.

In high-energy irradiated samples (Figure 2d,e) where *S*_e_ dominates, the appearance of uniform structures on the surface seems to be due to dewetting of Pt films. Since the kinetic sputtering of the film (dominated by high *S*_n_) is less in these two samples, well-isolated Pt islands are not visible on the surface. The large height variation on the surface as seen in the sectional analysis of the respective AFM images is due to Pt agglomeration (confirmed by the energy dispersive X-ray analysis) on the surface after ion irradiation. The sputtering of Pt and its re-deposition on the surface cannot result in such a uniform pattern on the surface. Therefore, transient thermal effects, activated by dewetting, are assumed to yield the uniform surface structures. These surface structures begin to disappear as the *S*_e_ decreases (see Figure 2c). In the sample irradiated with 50 keV (*S*_e_/*S*_n_ = 1), the surface structures disappear completely.

**Figure 3:**
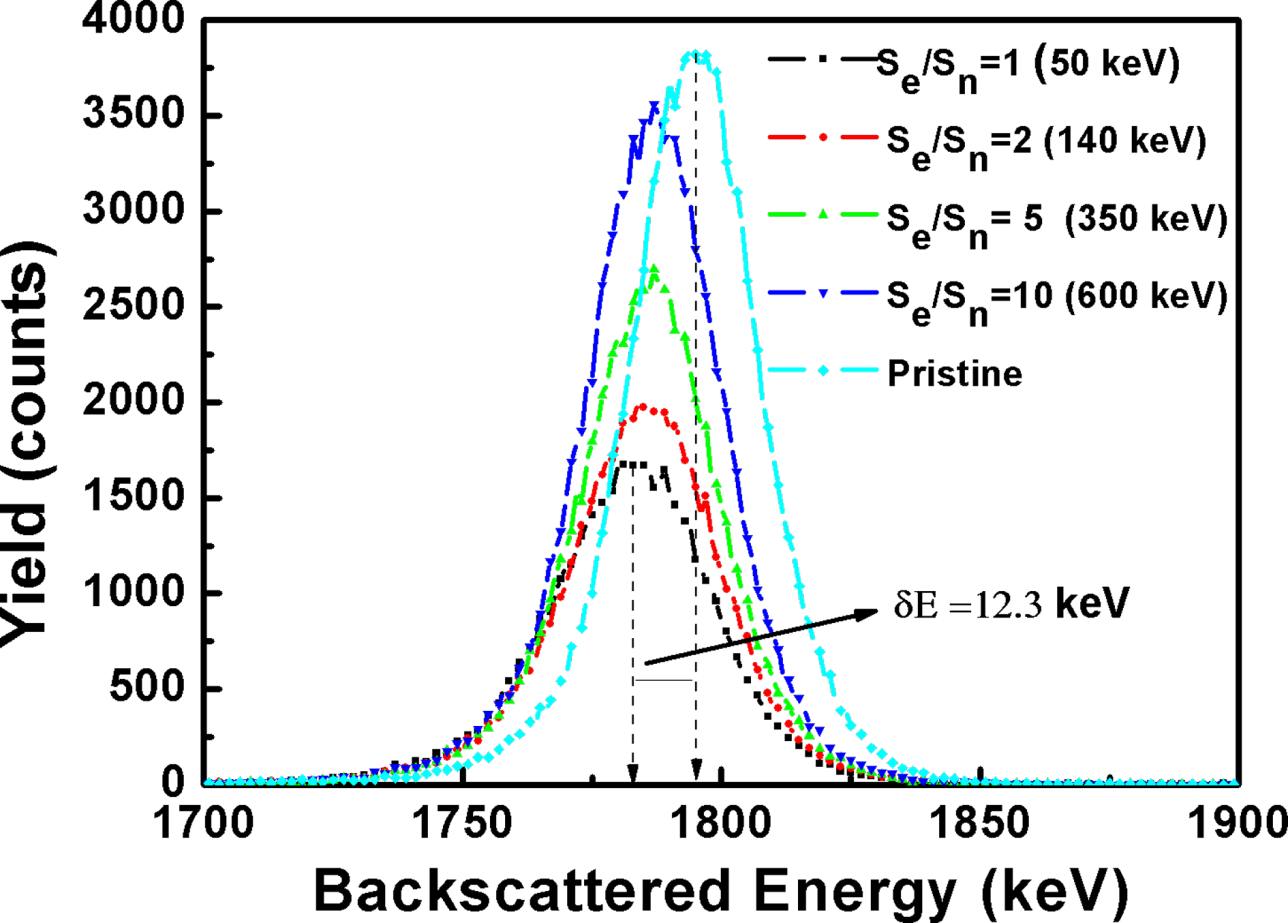
Rutherford backscattering spectra of the pristine and the irradiated films (Pt–Si).

Figure 3 shows the Rutherford backscattering spectra (at the Pt edge) of pristine and irradiated samples. The shifting of the Pt peak towards lower energy with a decrease in the *S*_e_/*S*_n_ ratio confirms the burrowing of Pt in Si. The Si edge (not shown here), however, remains unshifted due to the fact that energy loss by He^+^ ions in 5 nm thin Pt is negligible. The reduction in the height of the peak with decreasing *S*_e_/*S*_n_ shows the ion-induced sputtering of Pt. About a 50% Pt loss (area under the curve) is estimated in the film irradiated with 50 keV neon ions.

The Pt peaks for the different irradiation conditions are not fully resolved due to the extremely small thickness of the films. By taking the energy difference of the pristine Pt peak and the irradiated (*S*_e_/*S*_n_ = 1) film (12.3 keV), and utilizing the energy loss of the helium ions in the Pt–Si system as a function of the depth, ≈2 nm of burrowing was calculated in the irradiated film. If the elemental concentration is less than 10^14^ ions/cm^2^, detection with conventional RBS is difficult. In such a case, high-resolution, highly sensitive RBS measurements would be needed for accurate quantitative information on the burrowing.

**Figure 4:**
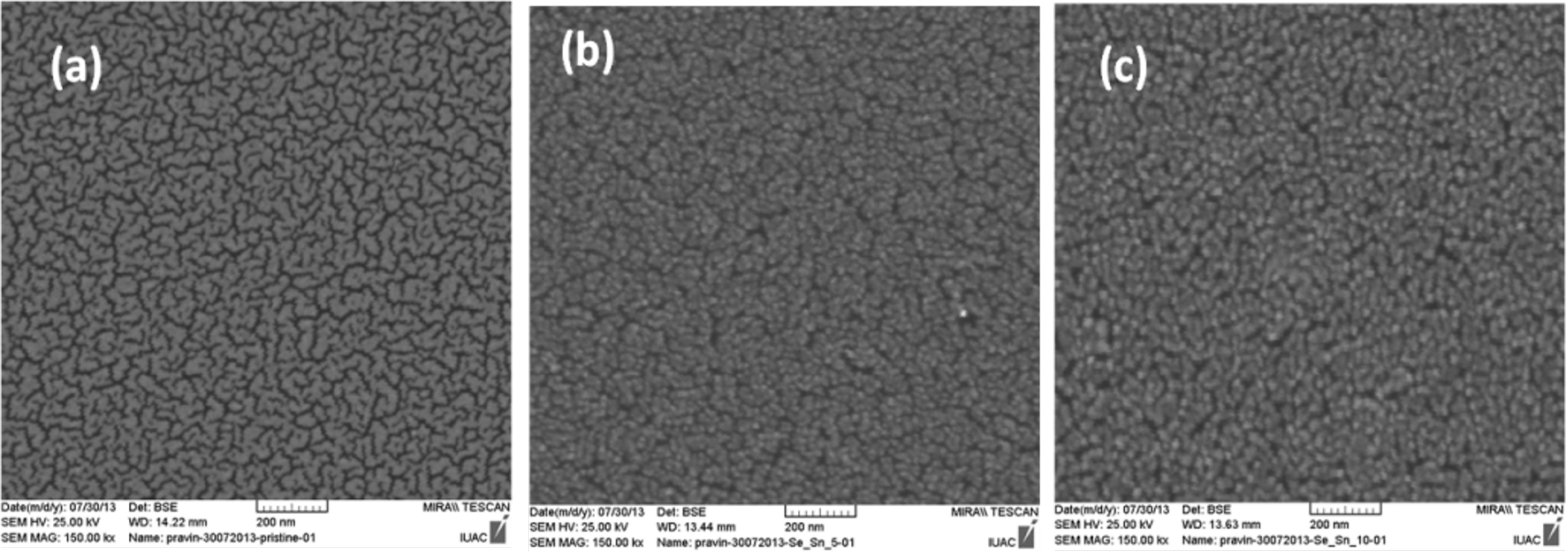
SEM images: a) pristine sample, b) 350 keV ion-irradiated film, and c) 600 keV ion-irradiated film.

The SEM images of the pristine and the ion-irradiated samples (only for 350 keV and 600 keV) are shown in Figure 4a–c. For the other samples irradiated with 140 keV and 50 keV energies, we could not get good contrast on the surface. As shown in Figure 4a, the Pt film on the Si is not very uniform. The formation of NPs (≈20 nm white spots in Figure 4c and <20 nm in Figure 4b) on the surface upon ion irradiation is confirmed. However, fade contrast in Figure 4b, in the sample irradiated with 350 keV, can either be due to the partial sinking of NPs in the substrate or due to the reduction in the size of the particles. SEM gives elemental information on the surface while AFM provides surface topography. Therefore, the features in the SEM and the AFM images cannot be compared quantitatively. Moreover, it is not possible to pinpoint the exact surface area by taking images with these two techniques. If surface structures are uniform, the correlation between the features governed by AFM and SEM can be discussed qualitatively. The Pt islands formed by kinetic sputtering followed by possible dewetting as seen in the AFM images (Figure 2d and 2e) are visible in the SEM images (Figure 4b and 4c) as white spots. The energy dispersive X-ray analysis shows a relatively larger atomic fraction of Pt when the electron beam is focused on these white spots. The discontinuous Pt film (pristine sample) as seen in the SEM image (Figure 4a) is also visible in the AFM image (Figure 2a) with certain grain size. The unusual heights at certain positions in the image (Figure 2a) may be due to dust particles on the surface.

**Figure 5:**
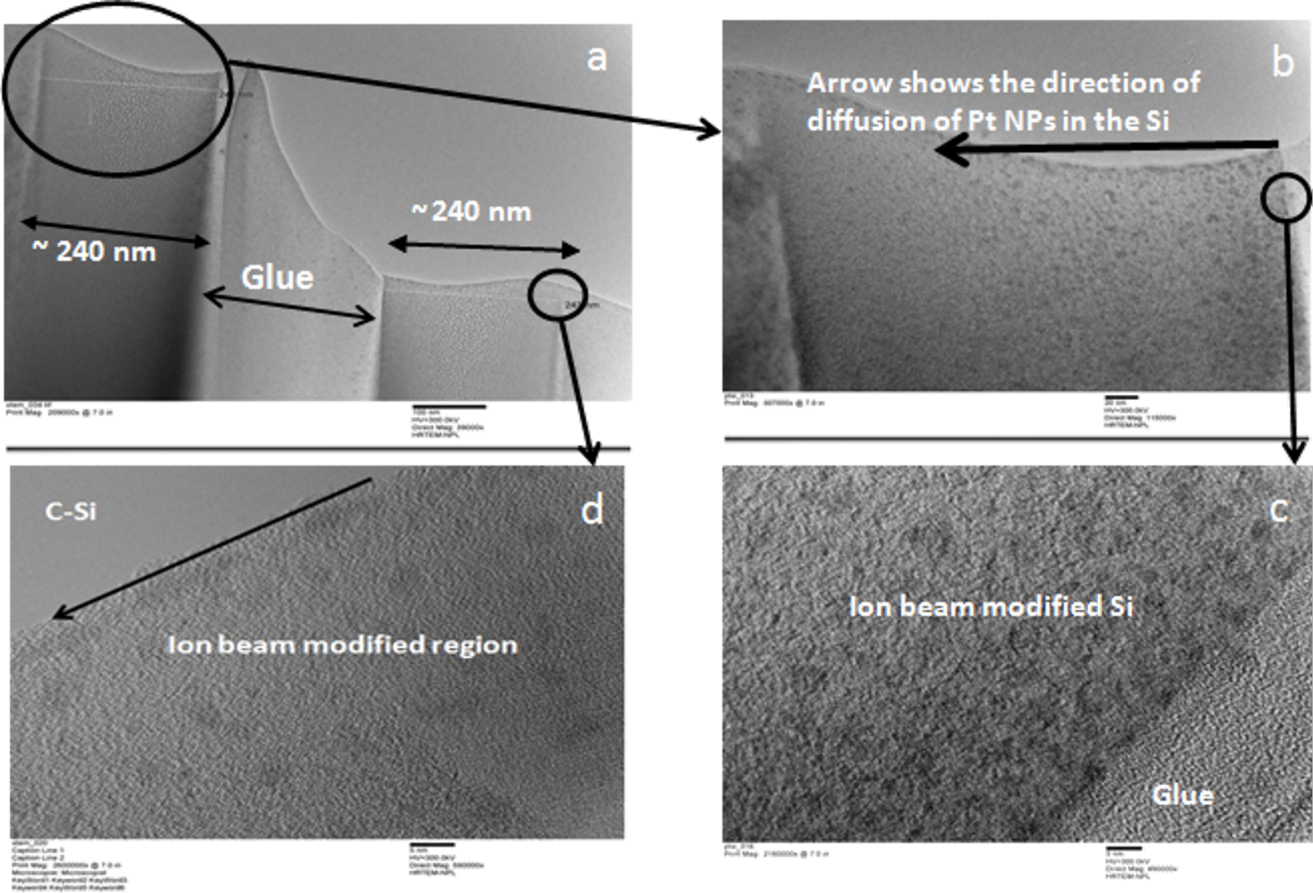
TEM images a) various interfaces, b) density distribution of NPs in ion beam modified region, c) interface showing high density of NPs near surface and absence of Pt on the surface, and d) NP distribution near the end of collision cascade.

**Figure 6:**
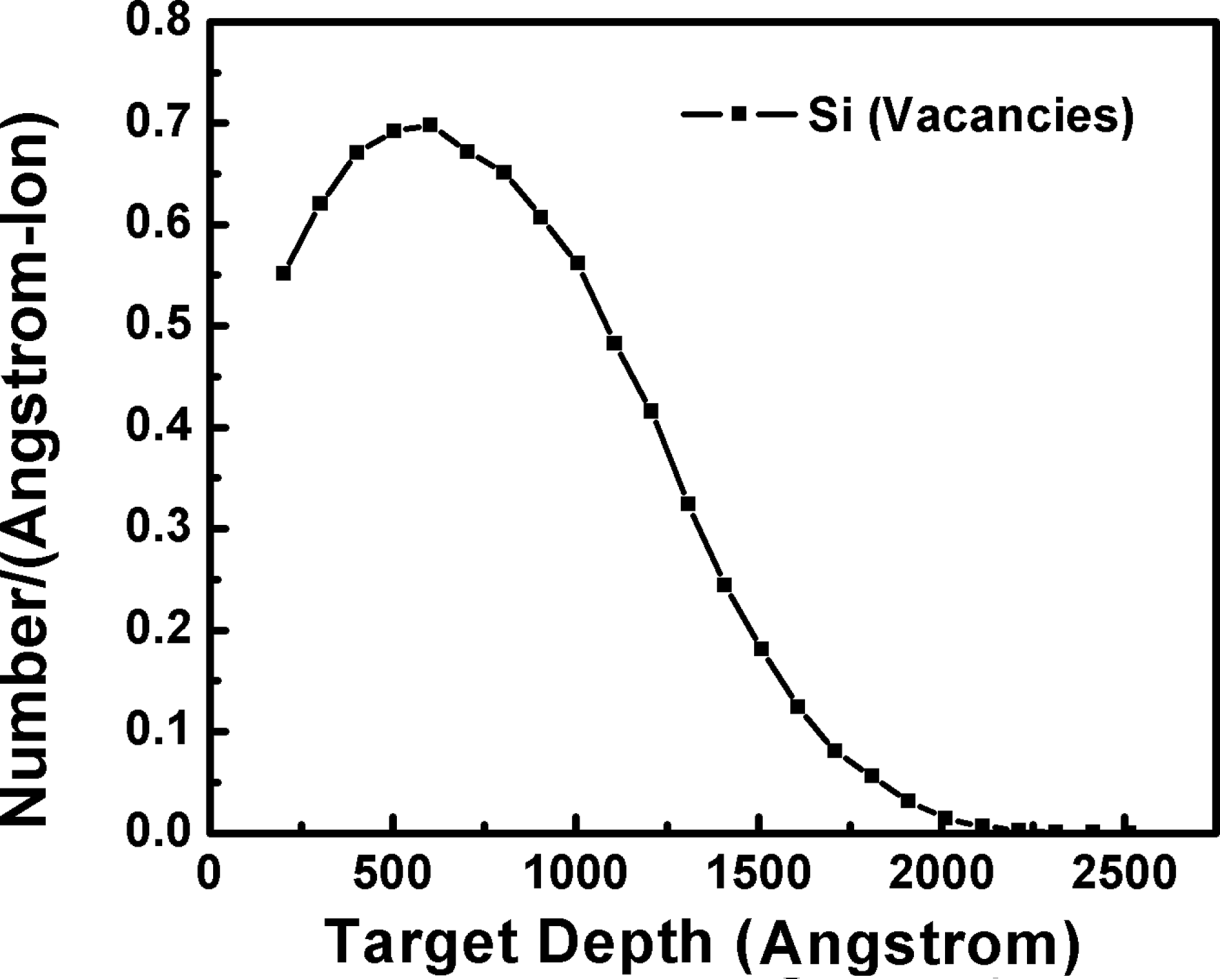
The distribution of silicon vacancies. The 50 keV neon ions were irradiated at normal incidence on 5 nm Pt film deposited on silicon substrate.

The high resolution XTEM analysis of one sample (irradiated with *S*_e_/*S*_n_ = 1) is shown in Figure 5. Figure 5a represents the cross sectional view of two silicon surfaces (cut from the same sample) joined face-to-face with glue (epoxy/adhesive substance). The range of 50 keV neon ions in Si is ≈107 nm with a longitudinal straggling of ≈46 nm. Therefore, one can expect a modified region of ≈130 nm below the surface upon ion bombardment. However, an interface at ≈242 nm below the surface is clearly seen in Figure 5a. This is mainly attributed to the amorphization of the silicon by collision cascade which can propagate even further than the range of ions (see Si vacancies profile distribution in Figure 6). Figure 5b, which is the zoomed image of the region covering the surface and the interface caused by the cascade (arrow moves from the surface to interface), shows that NPs are present up to the end of the collision cascade. However, the density of NPs decreases drastically with increasing depth (which could be less than the detection limit of the RBS within a few nm) and this is probably the reason why the Pt edge has a small shift (compared to pristine) in the RBS measurement of the sample. Figure 5c covers the near-surface region of HRTEM analysis which shows that ≈5 nm crystalline NPs are uniformly distributed below the surface. The density and the size distribution of NPs close to the end of collision cascade are shown in Figure 5d. The density of NPs decreases drastically. The size of NPs does not change much.

When varying the *S*_e_/*S*_n_ ratio, the maximum burrowing of Pt NPs was found for the films irradiated with 50 keV neon ions. Therefore, local defects (especially vacancies) produced by elastic collisions, which are governed by *S*_n_, are mainly responsible for the burrowing of NPs in silicon as also discussed by Hu et al. [22]. Given the irradiation parameters (50 keV energy, 1 µA beam current and 16 × 10^3^ s to irradiate 10^17^ ions/cm^2^ in a sample of 1 cm^2^ area) and the specific heat of silicon (710 J/kg∙K), we expected the target temperature to be at ≈500 K at the end of the irradiation [39]. The radiation and the heat conduction losses were not considered in the calculation. The diffusivity (*D*) of Pt in silicon at 500 K is ≈3 × 10^−19^ m^2^/s [40]. The total energy deposited (*F*_D_) by 50 keV ions in Si is ≈38 eV/Å. Given a target temperature of 500 K (*k*_B_*T* = 41 meV), an ion irradiation time, *t*, of 16 × 10^3^ s, and the relation *d* = *D* × *k*_B_*T* × *t*/*F*_D_ (where *k*_B_ is the Boltzmann constant), the diffusion length (*d*) of Pt into crystalline silicon is estimated to be ≈50 nm. Therefore, the presence of NPs beneath the surface and up to ≈250 nm is probably due to radiation-induced enhanced diffusion. Holm et al. [41] have reported Pt distribution into lightly damaged regions of silicon approximately congruous to the vacancies generated during implantation. At a typical fluence of 10^11^ ions/cm^2^, they observed an enhanced Pt accumulation approximately two orders of magnitude higher compared to diffusion in non-implanted silicon. At the very high fluence (10^17^ ions/cm^2^) in the present study, large vacancies produced by elastic collisions between ions and target atoms can give rise to an enhanced diffusion of Pt via a Frank–Turnbull mechanism [42], which requires a relatively low processing temperature. In Frank–Turnbull-type diffusion, impurity atoms/clusters (Pt in this case) move from the interstitial sites to the vacancies. The vacancy production in the materials during ion irradiation/implantation is linearly proportional to the ion fluence [43]. Therefore, enhanced diffusion of Pt via vacancy production by the ion irradiation at high fluence is quite possible. Furthermore, the silicon vacancy profile (TRIM calculation/simulation; shown in Figure 6) upon 50 keV neon ion irradiation, which seems to be responsible for the Pt diffusion, matches well with the NP distribution (obtained from the cross sectional HRTEM analysis) in the film irradiated under the same conditions. Total vacancies produced in the system for a chosen ion–target combination is 846/ion (TRIM calculations). Using the thermal properties of silicon (a specific heat of 710 J/kg∙K and a thermal conductivity of 150 W/m∙K) and electronic energy deposited by the ions in silicon, we expect a spike temperature of about ≈2540 K (within 1 ps and 1 nm away from the ion track) [44]. The melting point of silicon is ≈1400 K and transient molten zones (giving rise to viscous flow of Pt atoms) in silicon are possible by ion irradiation. Since the temperature spike quenches via electron–phonon coupling within 10^−11^ s, a very small contribution by the viscous flow in Pt diffusion is expected for entire irradiation time.

**Figure 7:**
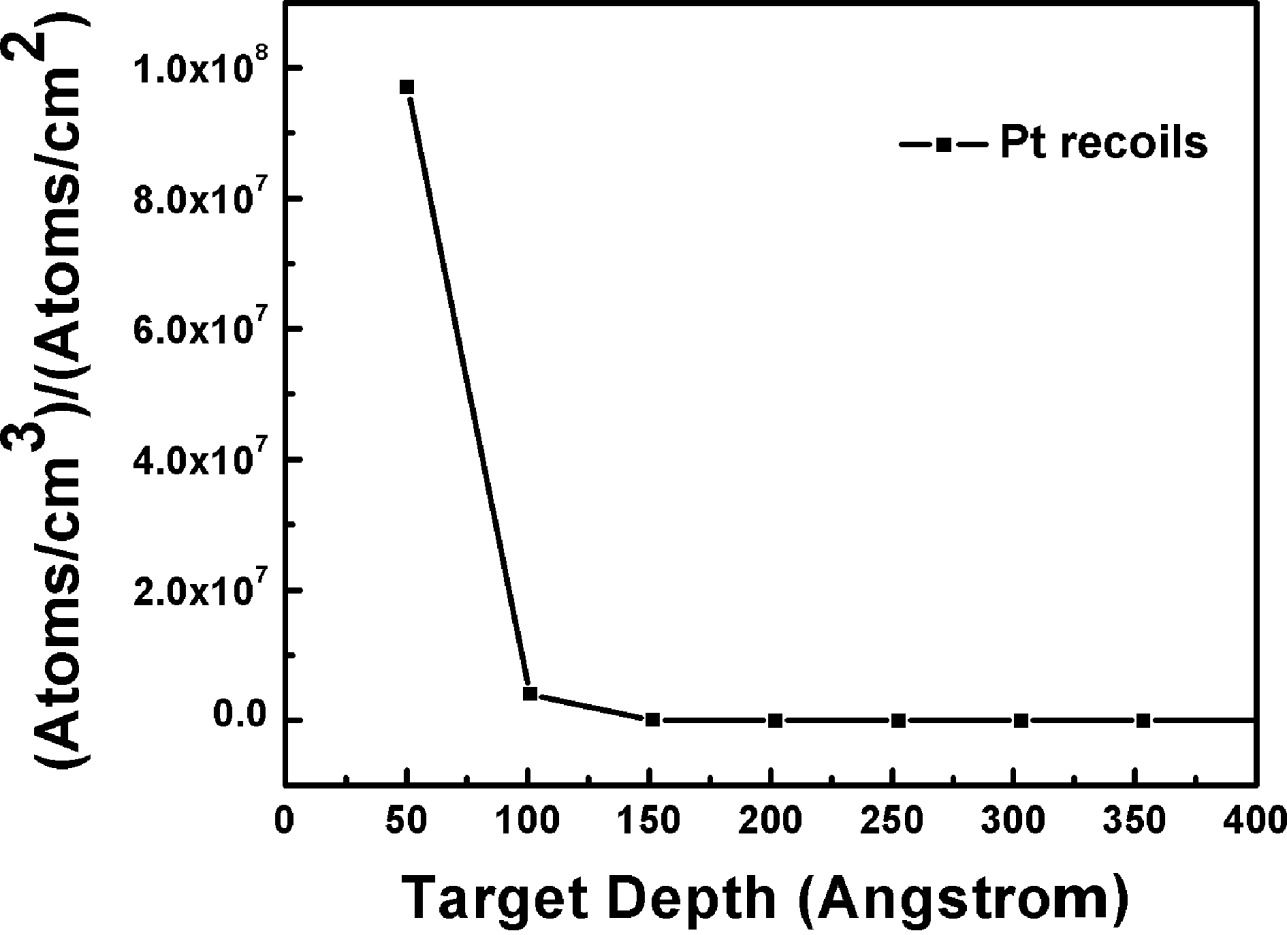
The distribution of Pt recoils (Pt/cm^3^ per Ne/cm^2^). The 50 keV neon ions were irradiated at normal incidence on 5 nm Pt film deposited on the silicon substrate.

Figure 7 shows the distribution of Pt recoils (TRIM calculation/simulation) for 50 keV neon ion irradiation on the Pt–Si system at normal incidence. The TRIM calculation takes an unperturbed system (point defects created by preceding ions are ignored) into account for each incident ion. The distribution shows that Pt atoms undergo near-surface recoil implantation upon ion irradiation.

**Figure 8:**
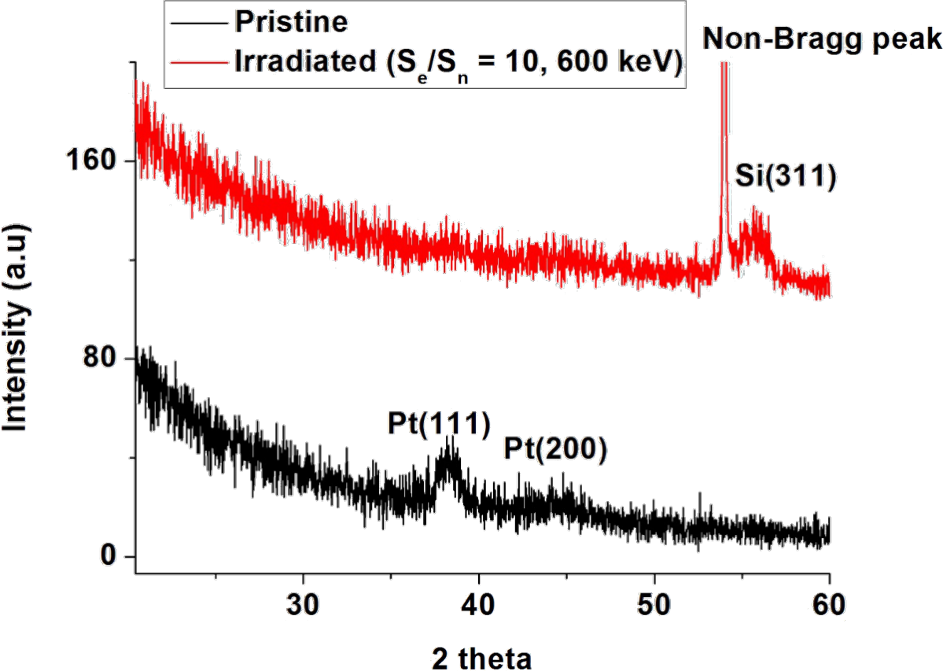
The XRD patterns of the pristine and the ion irradiated (*S*_e_/*S*_n_ = 10) films.

By observation of the uniform size of the Pt NPs (up to 240 nm beneath the surface), the density distribution of NPs (density decreases from surface to bulk), and the recoil distribution profile, it seems that the synthesis of the NPs takes place near the surface. The Pt NPs may then diffuse into the silicon via vacancies. Yet the question of the possible Pt NP formation mechanism for this experiment still remains. Considering the AFM and RBS measurement results (energy dependent changes in surface topography, shift and intensity loss in Pt peaks), we believe that the nuclear sputtering of the Pt film takes place during ion irradiation resulting in Pt islands on the Si surface. Transient thermal spikes generated by the ion beams are sufficient enough to melt the Pt islands. The spike temperature in Pt is expected to be ≈2000 K (within 10^−14^ s and 1 nm away from the ion trajectory) upon irradiation with 50 keV neon ions. In comparison to silicon, the temperature rise in Pt is faster due to its high electron density. The spike temperature in Pt is sufficient for the transient melting of islands (melting point of Pt is ≈1768 K). The molten Pt islands take a spherical shape to minimize their surface energy (dewetting). The surface energies of Pt and silicon are 2.49 and 1.51 J/m^2^, respectively. There could be electronic sputtering of these molten Pt islands giving rise to a uniform size of NPs on the surface. The recoiled Pt atoms underneath the surface may also agglomerate during thermal spikes giving rise to the satellite Pt NPs as reported in the literature [22,45]. The Ostwald ripening [46] of these satellite NPs can result in the final size (≈5 nm as seen by HRXTEM analysis) of NPs which undergo diffusion into the silicon. In Ostwald ripening, bigger clusters are grown at the expense of the dissolution of smaller cluster. The necessary temperature for this process is achieved through energy deposition from the ion beam. To confirm the formation of a silicide phase (if any) in irradiated films, the XRD patterns of pristine and ion-irradiated (only with *S*_e_/*S*_n_ = 10) films were recorded and are shown in Figure 8. The film irradiated with a high *S*_e_ is expected to undergo a phase formation due to a higher spike temperature along with the mingling of atoms by elastic collisions. Within the diffractometer detection limit, we could not find any silicide phase in the irradiated film and we believe that NPs (reported for film irradiated with *S*_e_/*S*_n_ = 1) consist only of Pt. We also expect absence of a silicide phase in the films irradiated with lower *S*_e_. The Pt film is polycrystalline in nature and (111) and (200) planes are clearly visible in the XRD pattern [47]. The reduced intensities and the broadening of the Pt peaks in the irradiated film confirm the Pt loss on the surface and the formation of NPs. The peak at around 2θ = 56º in the irradiated film is due to the Si substrate. The sharp feature just before Si substrate peak is a non-Bragg scattering peak.

## Conclusion

We have reported the synthesis and the burrowing of Pt NPs due to medium-energy neon ion irradiation for Pt thin films deposited on a silicon substrate (Pt–Si). The ion fluence was kept constant (10^17^ ions/cm^2^) during the irradiation. Several ion energies (50 keV, 140 keV, 350 keV and 600 keV) were chosen to vary *S*_e_/*S*_n_ (1, 2, 5 and 10) in the Pt–Si system. The synthesis of Pt NPs and their burrowing in Si was confirmed using AFM, SEM, XRD and RBS measurements. The relation between the energy losses of the ion beam and the synthesis and burrowing of Pt NPs are evidenced. The HRXTEM analysis of a single sample (irradiated with *S*_e_/*S*_n_ = 1) shows that the size of the NPs and the depth of the burrowing are ≈5 nm and ≈240 nm, respectively. Regarding the depth of the burrowing, the density of the NPs decreases drastically. The XRD analysis shows an absence of silicide phase within the detection limit of the instrument. Ion beam induced sputtering followed by partial dewetting of metallic films and recoil implantation seems to be the possible mechanism behind Pt NP (≈5 nm) formation. The ion-induced, silicon vacancy profile matches well with the Pt NP distribution underneath the surface. Therefore, radiation enhanced diffusion, in particular a Frank–Turnbull-type mechanism, is likely responsible for the large diffusion (≈240 nm deep) of Pt NPs into the silicon.

## Supporting Information

File 1 Additional information.
