# Supplementary material for: Synthesis of Pt nanoparticles and their burrowing into Si due to synergistic effects of ion beam energy losses
Source: Beilstein J Nanotechnol. 2014 Oct 24;5:1864–72. doi: 10.3762/bjnano.5.197 (PMC4222290; doi:10.3762/bjnano.5.197)

Supporting Information

for

Synthesis of Pt nanoparticles and their burrowing into Si due to synergistic effects of ion beam energy losses

Pravin Kumar[1*], Udai Bhan Singh[1], Kedar Mal[1], Sunil Ojha[1], Indra Sulania[1], Dinakar Kanjilal[1], Dinesh Singh[2] and Vidya Nand Singh[2]

Address: [1]Inter University Accelerator Centre (IUAC), New Delhi 110067, India and [2]National Physical Laboratory (NPL), New Delhi 110012, India

Email: Pravin Kumar - vishakhapk@gmail.com

* Corresponding author

Additional information

Spectrum processing :

Peaks possibly omitted : 3.489, 15.650 keV

Processing option : All elements analyzed (Normalised)

Number of iterations = 3

Standard :

Si SiO2 1-Jun-1999 12:00 AM

Pt Pt 1-Jun-1999 12:00 AM

Element	Weight%	Atomic%
Si K	99.55	99.94
Pt M	0.45	0.06
Totals	100.00	

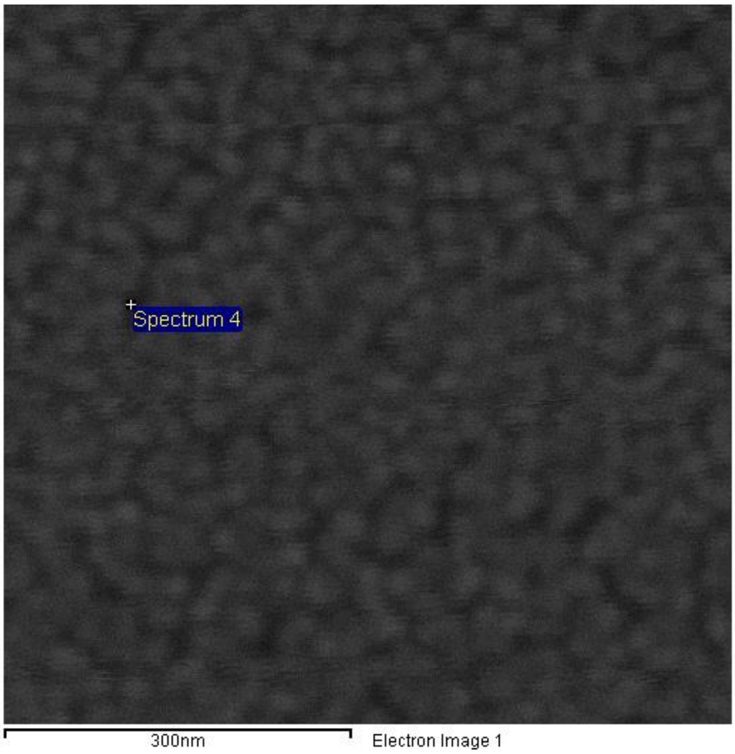

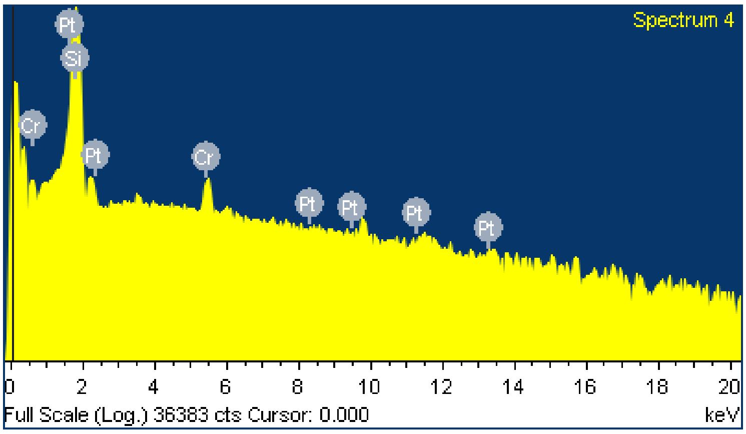

Spectrum processing :

Peak possibly omitted : 3.488 keV

Processing option : All elements analyzed (Normalised)

Number of iterations = 3

Standard :

Si SiO2 1-Jun-1999 12:00 AM

Pt Pt 1-Jun-1999 12:00 AM

Element	Weight%	Atomic%
Si K	99.25	99.89
Pt M	0.75	0.11
Totals	100.00	

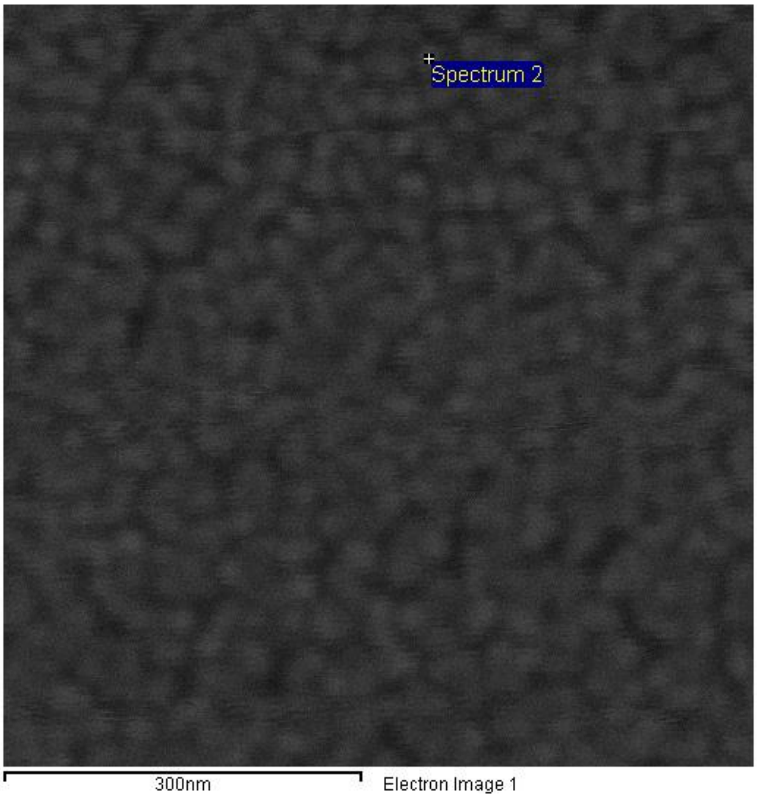

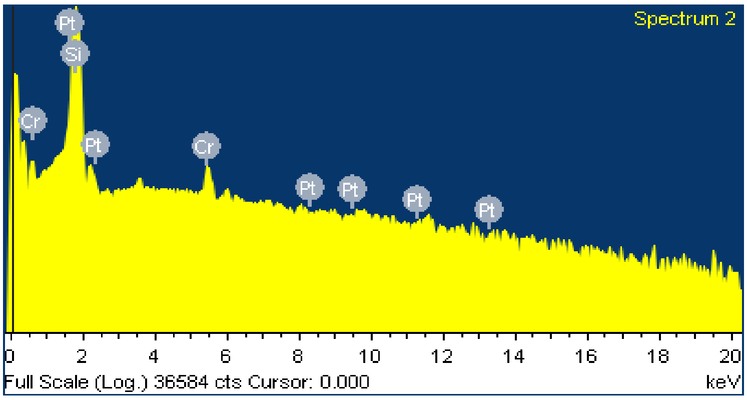

Supplement: File 1 — Additional information. [file Beilstein_J_Nanotechnol-05-1864-s001.pdf]
